# Supplementary material for: Selective clearance of aberrant tau proteins and rescue of neurotoxicity by transcription factor EB
Source: EMBO Mol Med. 2014 Jul 28;6(9):1142–60. doi: 10.15252/emmm.201303671 (PMC4197862; doi:10.15252/emmm.201303671)
Supplement: Supplementary file 1 — Supplementary Figures [file emmm0006-1142-SD1.pdf]

Contents:

Figure S1. P0 injection of AAV-GFP in rTg4510 and wild-type mice infected neurons in the cortex and hippocampus in a wide spread manner.

Figure S2. TFEB has no observed effect on APP expression or Aβ pathology.

Figure S3. Immunofluorescence analysis showing aberrant Tau pathology being greatly reduced by AAV-TFEB injection in the hippocampus of rTg4510 mice.

Figure S4. Intracellular clearance of pTau pathology by AAV-TFEB injected to adult mice brains.

Figure S5. Gene set enrichment analysis (GSEA) of transcriptome changes of synaptic genes by TFEB treatment .

Figure S6. Analysis of lysosomal and autophagy genes as a function of TFEB expression in wild-type and Tau mice.

Figure S7. Autophagic flux is increased by TFEB overexpression.

Figure S8. Phospho-tau (pTau) is observed to associate with both autophagic vesicles and lysosomes in vitro.

Figure S9. AAV-TFEB injection mildly increase PTEN at protein level in vivo.

Figure S10. TFEB mediates Akt-mTOR signaling pathway in correlation with PTEN upregulation.

Supplementary Figure 1

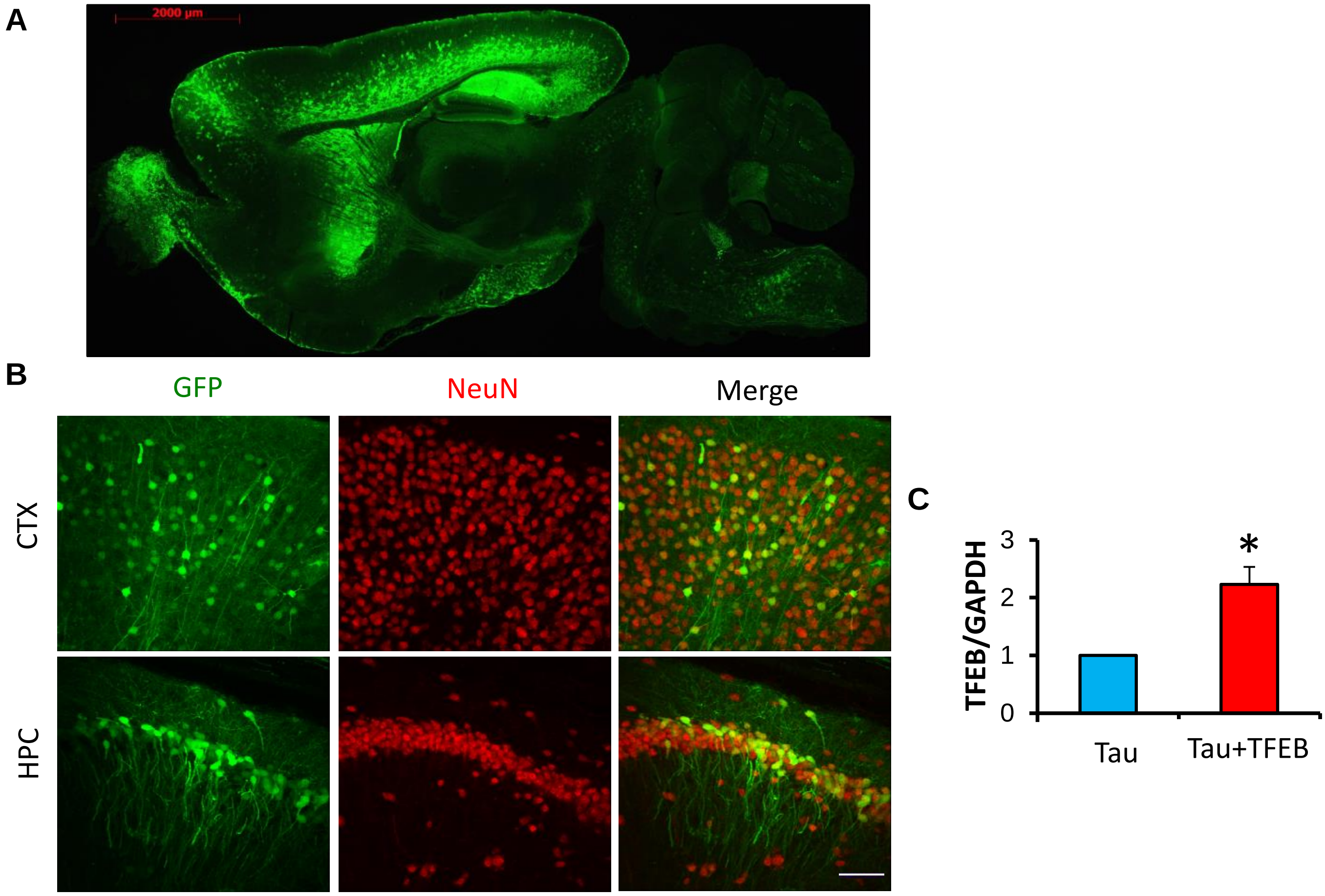

Figure S1. P0 injection of AAV-GFP in rTg4510 and wild-type mice infected neurons in the cortex and hippocampus in a widespread manner. A. Representative sagittal sections of mice injected with AAV-GFP. Scale bar: 2000 µm. B. AAV-GFP P0 injected mice were analyzed at four months (n=4). Sections underwent immunofluorescence staining using antibodies for GFP and NeuN. Scale bar= 50 µm. C. qRT-PCR analysis of TFEB expression in rTg4510 Tau mice (Tau) or Tau mice injected with AAV-TFEB (Tau + TFEB). N=3 mice/group in triplicates. Each bar represents average ± s.e.m.

Supplementary Figure 2

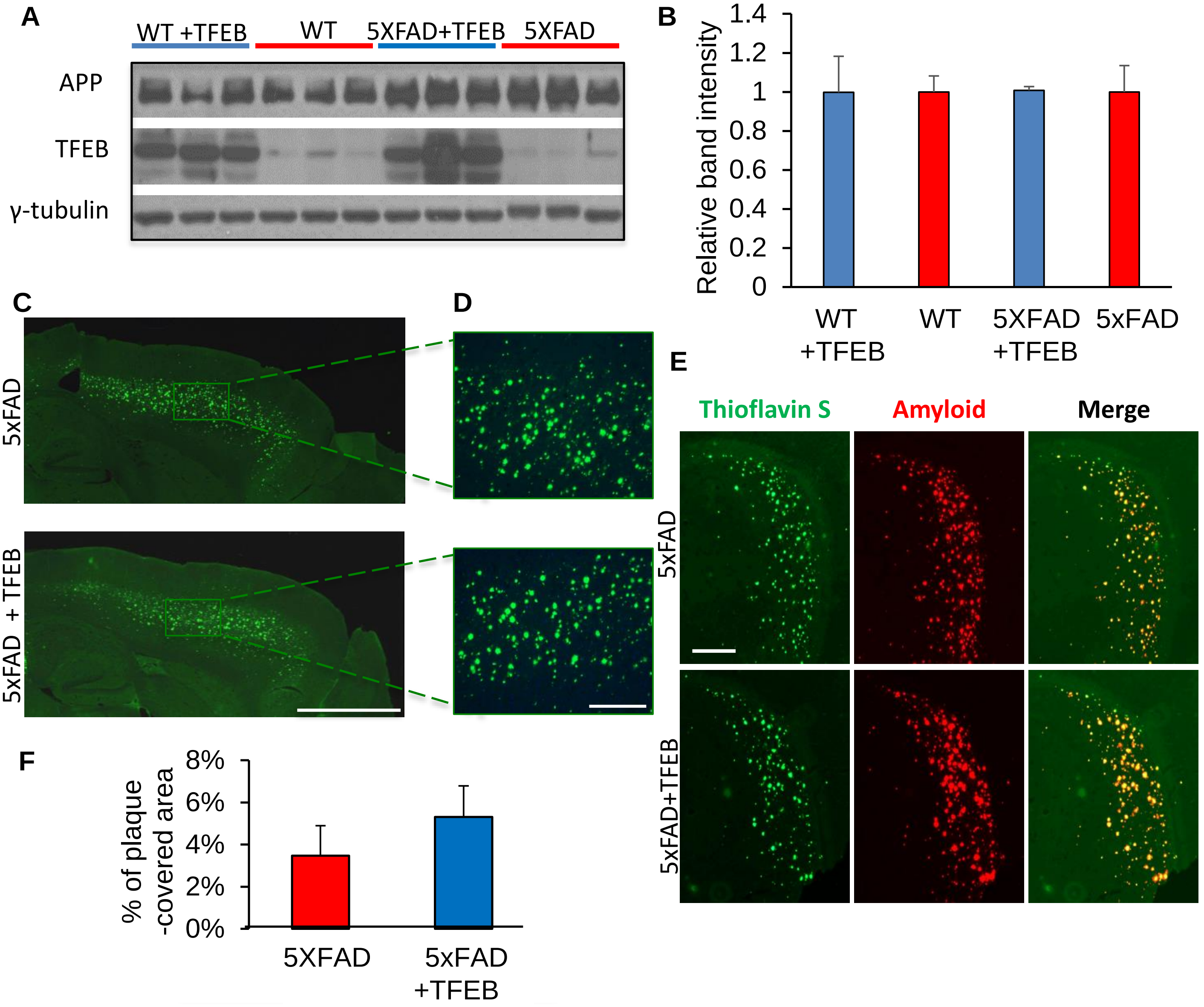

Figure S2. TFEB has no observed effect on APP expression or Aβ pathology. A. Western blot analysis showing TFEB P0 injection has not changed expression level of APP in 5XFAD mice when the animals were analyzed at 2 month of age. B. Quantification of APP western blot in A. C. and D. Representative low (C) and high (D) resolution images of amyloid pathology detected by 6E10 antibody in brains of 5xFAD APP or APP+TFEB mice. E. Immunofluorescent images showing signals from the amyloid antibody (used in F for plaque load quantification) colocalized well with Thioflavin S staining. F. Quantification of Aβ load in the cortical area. N= 5mice/group/experiment. Scale bars in C, D, and E are: 2000 μm, 200 μm, and 400 μm, respectively. Each bar represents average ± s.e.m.

Supplementary Figure 3

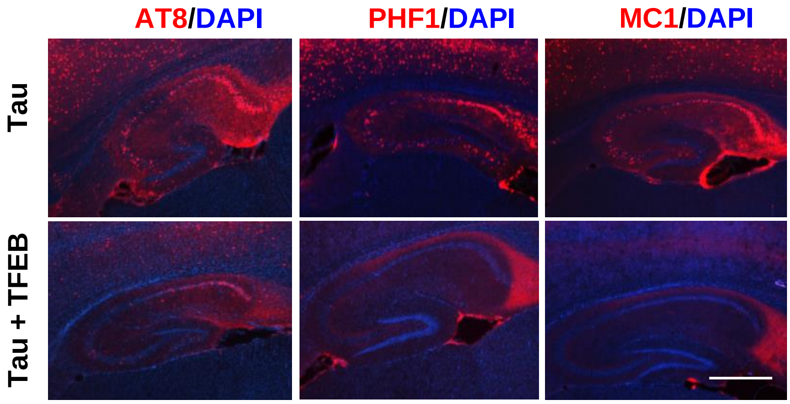

Figure S3. Immunofluorescence analysis showing aberrant Tau pathology being greatly reduced by AAV-TFEB injection in the hippocampus of rTg4510 mice. AT8, PHF1 or MC1 antibodies were used and sections were countered with DAPI. Scale bar: 1000 µm. N=5 mice/treatment group.

Supplementary Figure 4

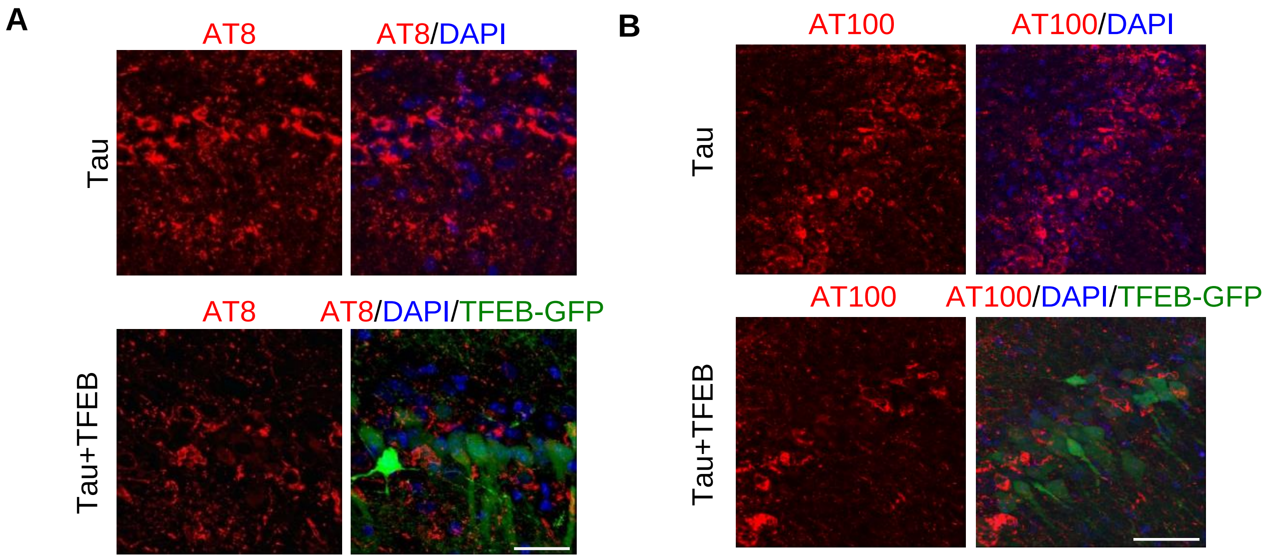

Figure S4. Intracellular clearance of pTau pathology by AAV-TFEB injected to adult mice brains. A. Immunohistochemical staining of hippocampus of rTg4510 (Tau) mice or Tau mice co-injected with AAV-TFEB/AAV-GFP (Tau + TFEB) at 2 months and analyzed at 4 months. Left panels: AT8 only; Right panels: Merged images of AT8/DAPI (top) or AT8/DAPI/GFP (bottom), showing that TFEB-GFP-positive neurons are negative for AT8 staining. B. Immunofluorescence staining using AT100 (T212/S235) antibody in CA1 region neurons of the hippocampus of the same group, showing lack of hyperphosphorylated Tau at T212/S235 in TFEB/GFP positive neurons. Scale bar: 50 µm. N=5 mice/treatment group.

Supplementary Figure 5

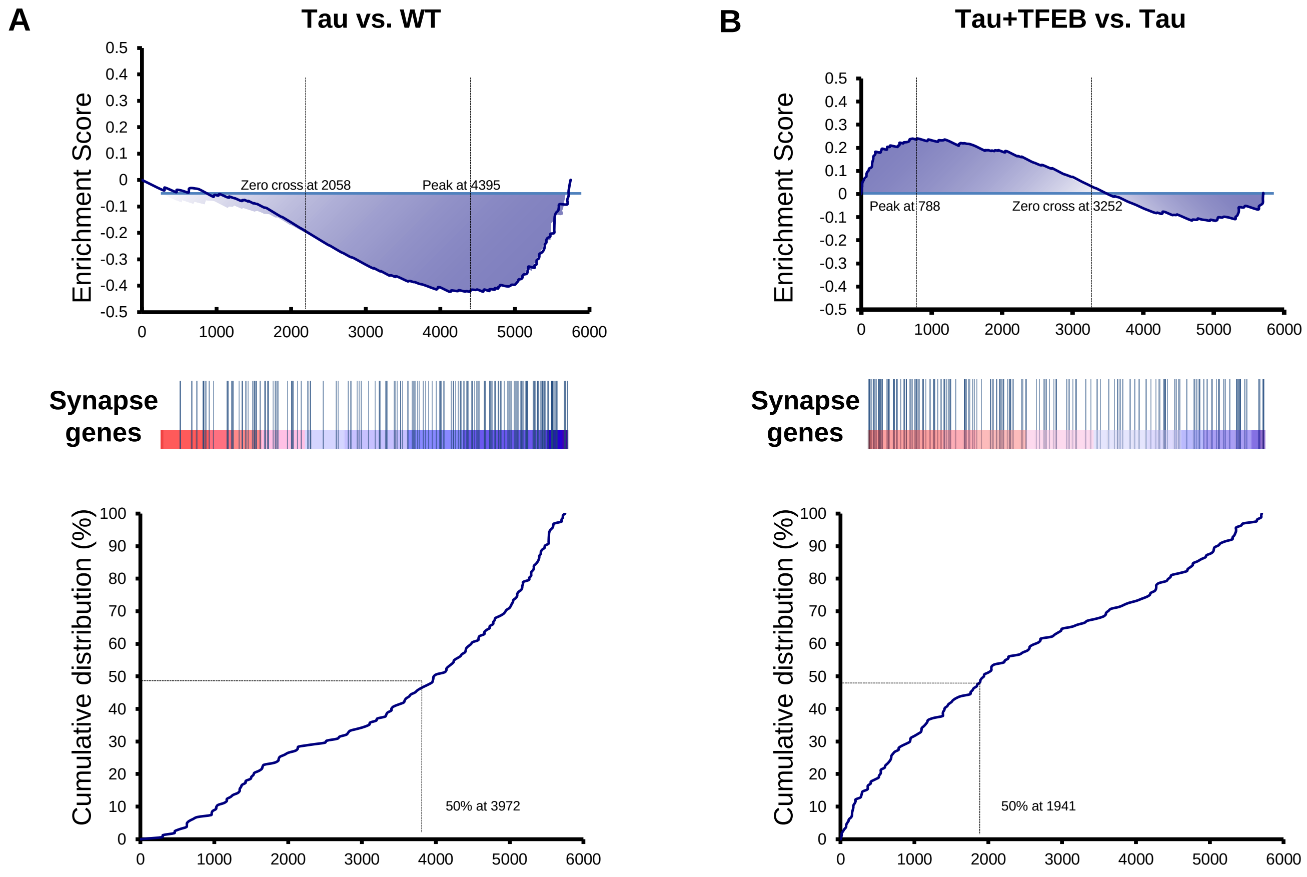

Figure S5. Gene set enrichment analysis (GSEA) of transcriptome changes of synaptic genes by TFEB treatment. GSEA of transcriptome changes of Tau mice compared with wild-type mice (A) or TFEB-injected Tau mice compared with uninjected Tau mice (B). GSEA of genes annotated as participating in the synaptic function are reported. Upper panels show enrichment generated by GSEA of ranked gene expression data (left in upper panel and red in middle panel: upregulated; right in upper panel and blue in middle panel: downregulated). The enrichment score is shown as a blue line. In the middle panels, vertical blue bars indicate the position of synapse genes within the ranked lists. Lower panels show the cumulative distribution of synapse genes within the ranked lists. The ranking positions that include 50% of synapse genes are indicated. The analysis shows that synapse genes tend to be downregulated in Tau mice compared with wild-type littermates (ES = -0.43, $P < 0.001$) (A), an effect that is mitigated by TFEB injection as shown by the comparison with non-injected Tau mice (ES = 0.24, $P < 0.001$) (B). N=4 mice/genotype/treatment group.

Supplementary Figure 6

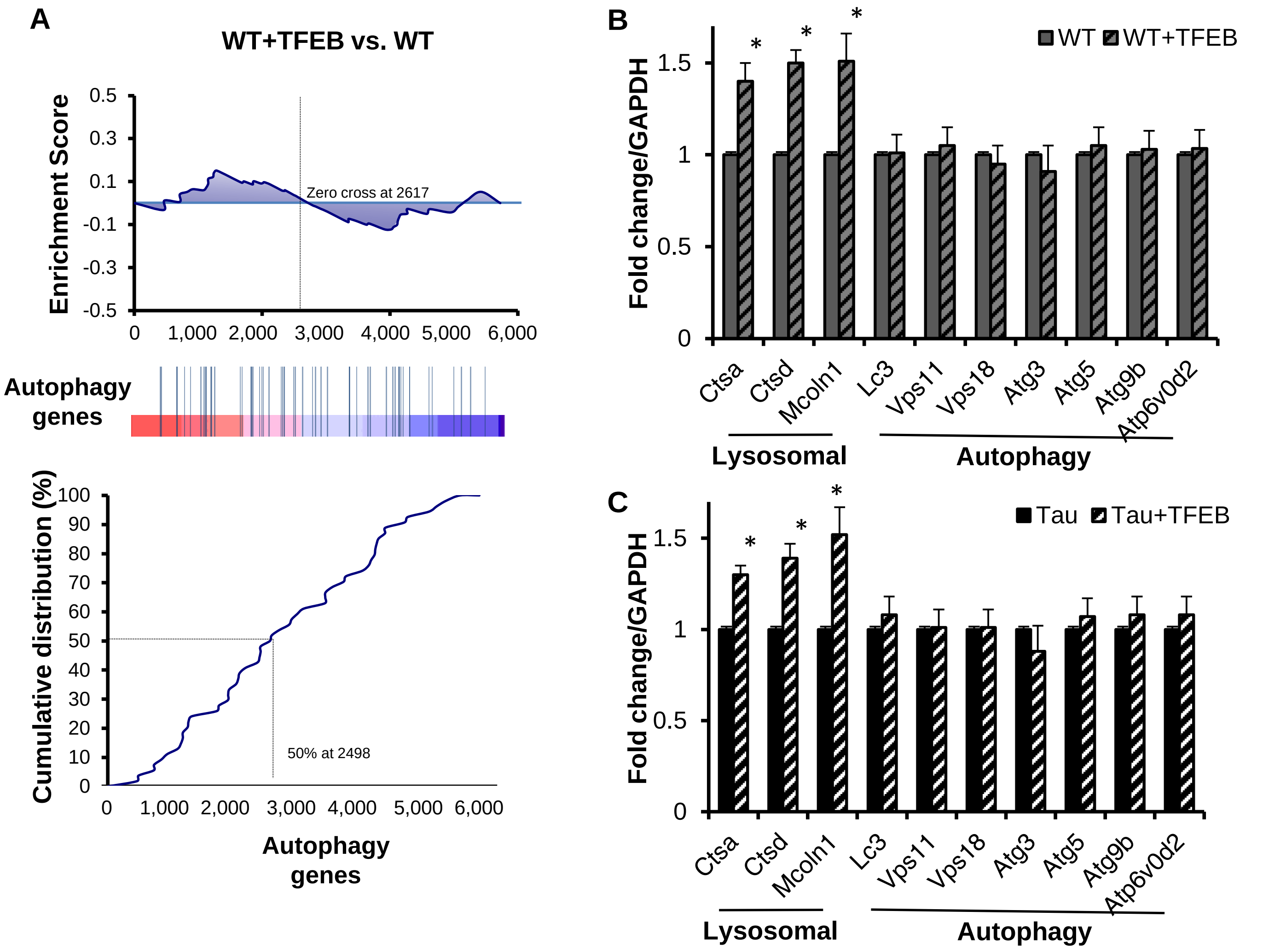

Figure S6. Analysis of lysosomal and autophagy genes as a function of TFEB expression in wild-type and Tau mice. A. Gene set enrichment analysis (GSEA) of transcriptome changes in TFEB-injected vs. uninjected wild-type (WT + TFEB vs. WT) mice of autophagy genes. The same analysis as Figure 6A is performed except that autophagy gene expression data was used. The analyses show that autophagy genes do not have a significant global shift towards upregulated or downregulated genes in TFEB-injected wild-type mice compared with uninjected littermates (ES = 0.08, $P > 0.99$). N=4 mice/group. Lower panel of A shows cumulative distribution of autophagy genes within the ranked list. The ranking positions that include 50% of autophagy genes (ES = 0.08, $P > 0.99$) are indicated. N=4 mice/genotype/treatment group. B. and C. qRT-PCR analysis of selected TFEB lysosomal or autophagy target genes as indicated in TFEB-injected vs. uninjected wild-type (B) or Tau (C) mice. N=3 mice/genotype/treatment group each in triplicates. Values are average ± s.e.m. *, $p<0.05$ (Student *t*-test). Oligonucleotide sequences are as follows: Ctsa-F: CCCTCTTTCCGGCAATACTCC; Ctsa-R: CGGGGCTGTTCTTTGGGTC
Ctsd-F: GCTTCCGGTCTTTGACAACCT; Ctsd-R: CACCAAGCATTAGTTCTCCTCC
MCOLN1-F: GCGCCTATGACACCATCAA; MCOLN1-R: TATCCTGGCACTGCTCGAT
LC3-F: TTATAGAGCGATACAAGGGGGAG; LC3-R: CGCCGTCTGATTATCTTGATGAG
VPS11-F: GGAGCCTGGTCTTTGGAGA; VPS11-R: GCTGTAGAGAACGTGGCAAGA
Vps18-F: TTGCAGACTGGTTGCCCTA; Vps18-R: TTCTCCAGGTGGGCACTTAC
Atg3-F: GGGTGTAATCACCCCAGAAG; Atg3-R: TGTTGGACAGTGGTGGACTAA
Atg5-F: AAGTCTGTCCTTCCGCAGTC; Atg5-R: TGAAGAAAGTTATCTGGGTAGCTCA
Atg9b-F: GGGACATCCAGGTGTTTTACA; Atg9b-R: TTCCAGGAGGCGAGACTG
Atp6vod2-F: AAGCCTTTGTTTGACGCTGT; Atp6vod2-R: GCCAGCACATTCATCTGTACC
PTEN-F: TTTGCTAGTGAGTGGAATCCTCT; PTEN-R: TGTGACAAAAGTGACACAGATCA
GAPDH-F: TGCACCACCAACTGCTTAGC; GAPDH-R: GGCATGGACTGTGGTCATGAG

Supplementary Figure 7

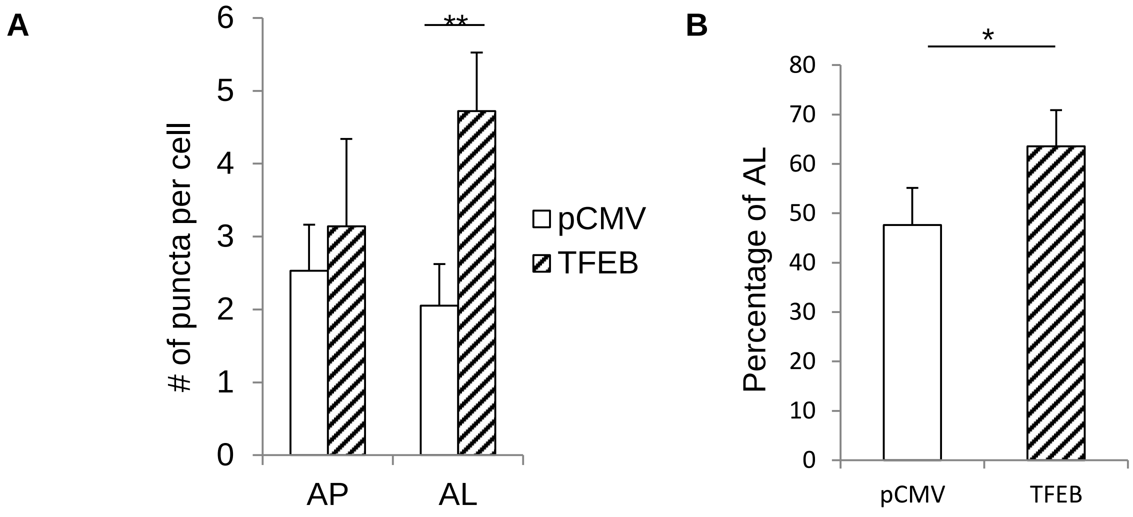

Figure S7. Autophagic flux is increased by TFEB overexpression. A. Quantification of the number of autophagosome (AP) and autolysosome (AL) per cell in TFEB overexpression and control cells. **, p = 0.003511. B. Percentage of AL in TFEB overexpression and control cells. *, p= 0.04286. N of pCMV = 47, n of TFEB = 33. Each bar represents average ± s.e.m.

Supplementary Figure 8

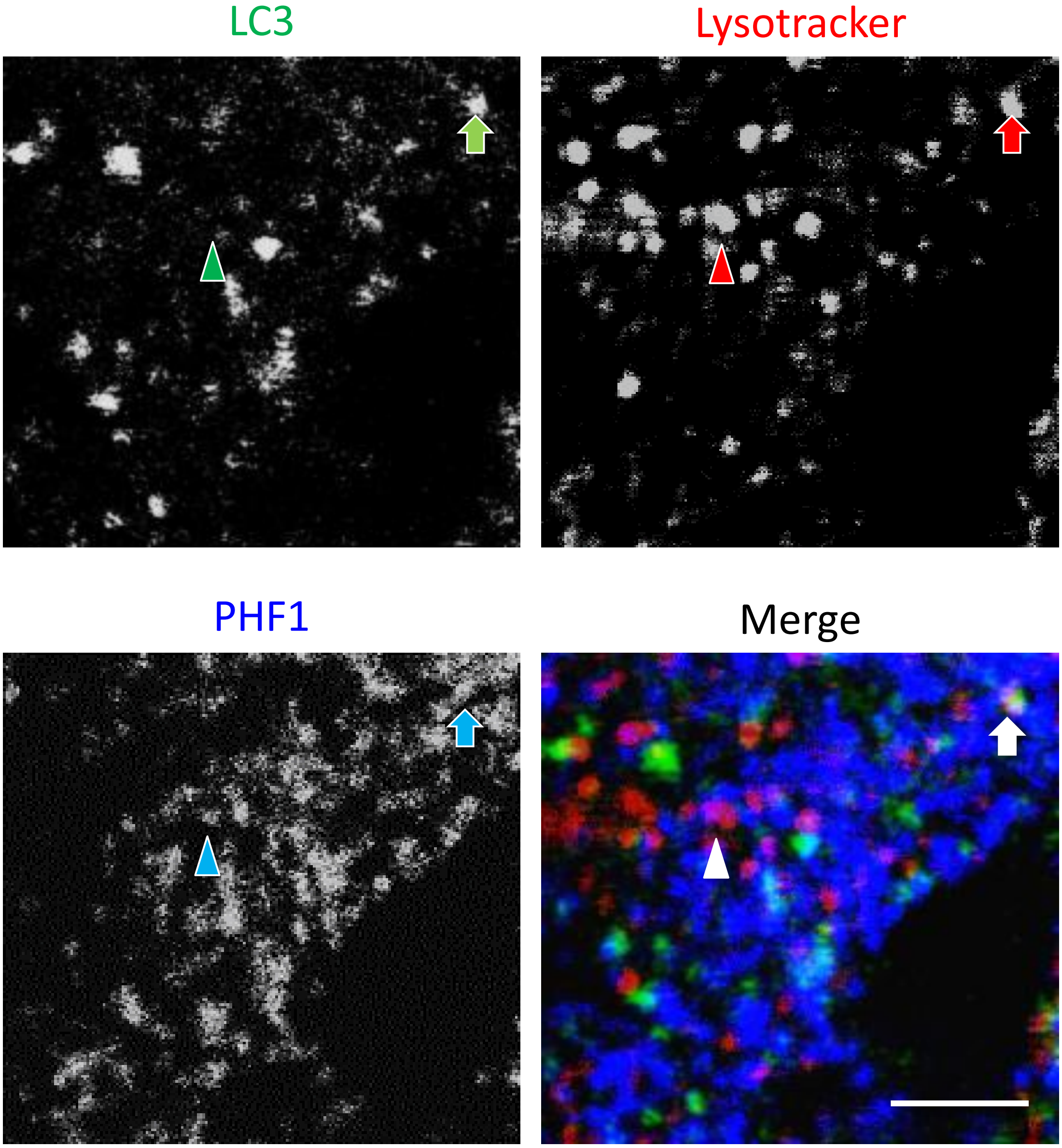

Figure S8. Phospho-tau (pTau) is observed to associate with both autophagic vesicles and lysosomes in vitro. pTau expression was induced in Tau40PL cell line by 1 μg/ml Doxycycline in the culture medium for 48 hrs. 50 uM of lysotracker-Red was added to the culture medium 30 minutes before the cells were fixed for immunoflurescent study. Arrow head in the above confocal image indicates a purple a punctum, which means colocalization of PHF1 (blue) and lysotracker-red, suggesting phospho-tau being targeted to lysosome. Puncta with cyan color are colocalization of LC3 (green) and PHF1 (blue), suggesting phospho-tau's involvement with autophagic vesicles. Arrow points to a white puncta, indicating colocalization of PHF1 (blue), lysotracker-red and LC3 (green), suggesting that autophagosome delivered phospho-tau into lysosome.

Supplementary Figure 9

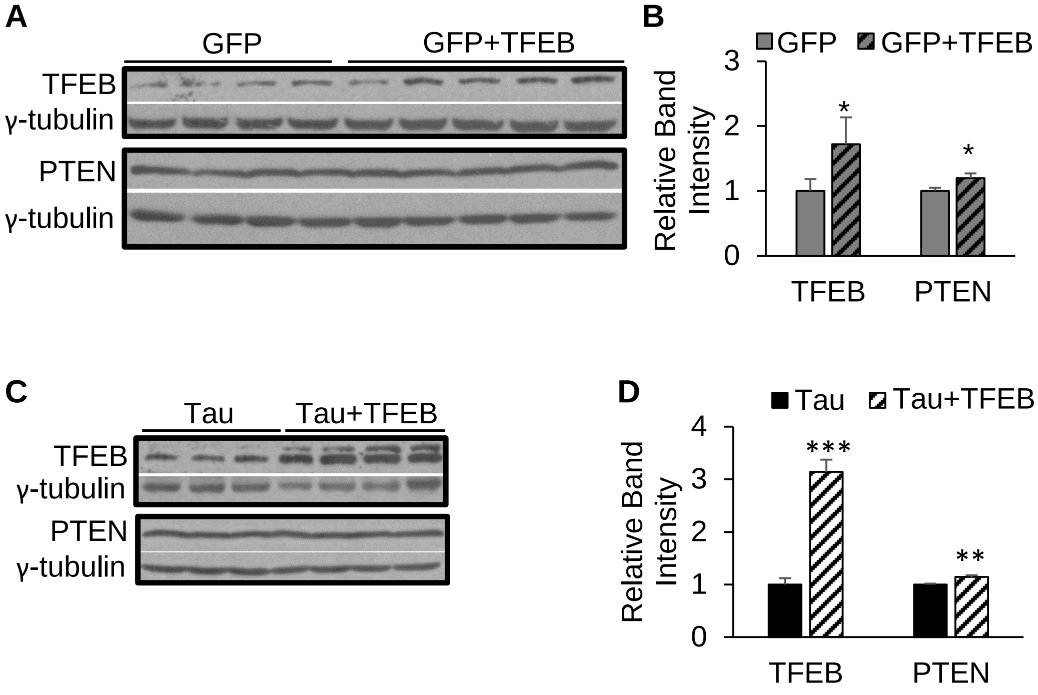

Figure S9. AAV-TFEB injection mildly increase PTEN at protein level in vivo. A. Western blot analysis of TFEB and PTEN expression in 1 month old P0 TFEB-FLAG/GFP (GFP + TFEB) and GFP only (GFP) injected wild-type mice. B. Quantification of relative band intensities of (B). PTEN is increased by 19% in the TFEB injected group, quantified by ImageJ. N=4 and 5 per group. TFEB protein levels are significantly increased in GFP+TFEB mice vs GFP only mice (P=0.046) as are PTEN protein levels (P=0.013). C. Western blot analysis of TFEB and PTEN expression in Tau mice with (+TFEB) or without AAV-TFEB adult injection. D. Quantification of relative band intensities of (C). PTEN is increased by 14% in the TFEB injected group, quantified by ImageJ. N=3 and 4 per group. TFEB protein levels are significantly increased in Tau+TFEB vs Tau (P=0.00013) as are PTEN protein levels (P=0.021) (Student's t-test). Each bar represents average ± s.e.m.

Supplementary Figure 10

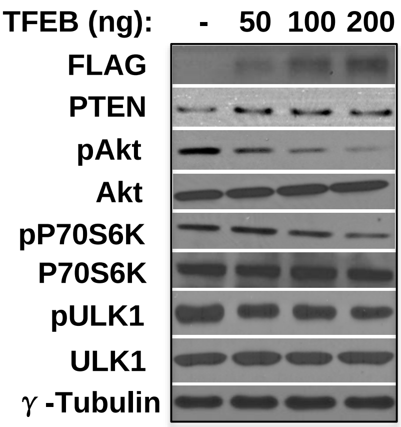

Figure S10. TFEB mediates Akt-mTOR signaling pathway in correlation with PTEN upregulation. TFEB-3xFLAG plasmid was tranfected into N2A cells at different dosages per well for 24-well plate. 0 ng TFEB dosage was tranfected with 200ng of pCMV5 plasmid as control. Western blot analysis of TFEB-FLAG, PTEN, total and phospho-Akt (S473), total and phospho-P70S6K (T389), and total and phospho-ULK1 (S757) levels in response to increasing amount of TFEB expression. γ-tubulin was used as a loading control. The experiment was done three times each in triplicates.
