# Supplementary figures and images for: Selective clearance of aberrant tau proteins and rescue of neurotoxicity by transcription factor EB
Source: EMBO Mol Med. 2014 Jul 28;6(9):1142–60. doi: 10.15252/emmm.201303671 (PMC4197862; doi:10.15252/emmm.201303671)

# Supplementary Figure 2 A

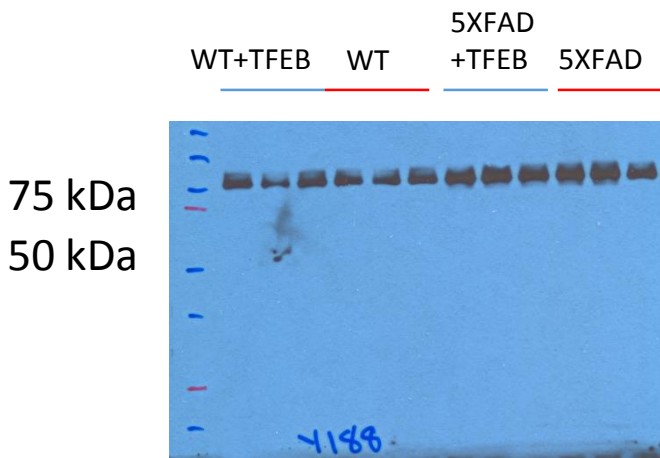

APP

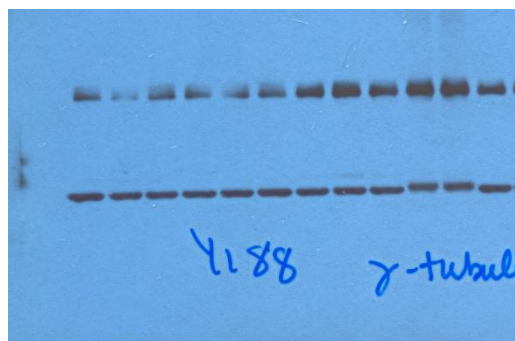

$\gamma$ -tubulin

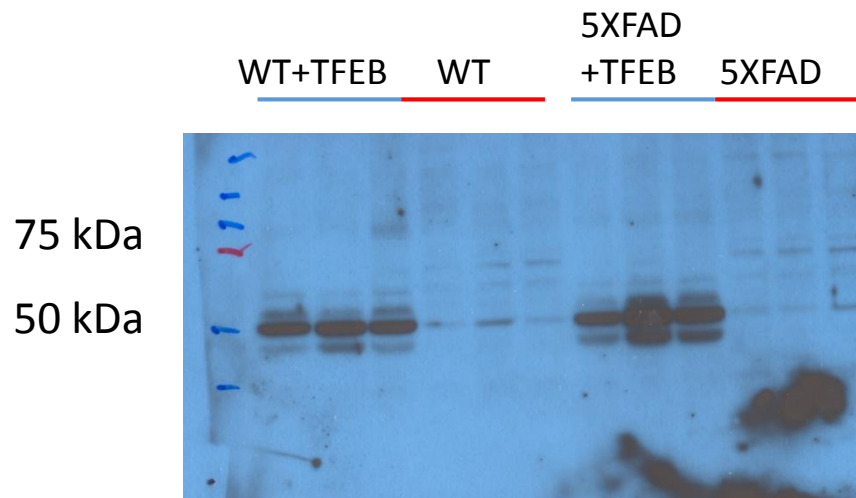

TFEB

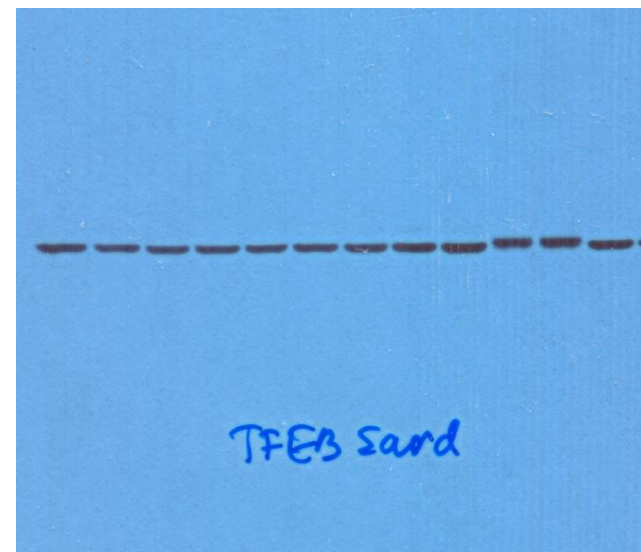

$\gamma$ -tubulin

Supplement: Supplementary file 2 — Source data for Supplementary Figure S2 A [file emmm0006-1142-SD2.pdf]

# Supplementary Figure 9A

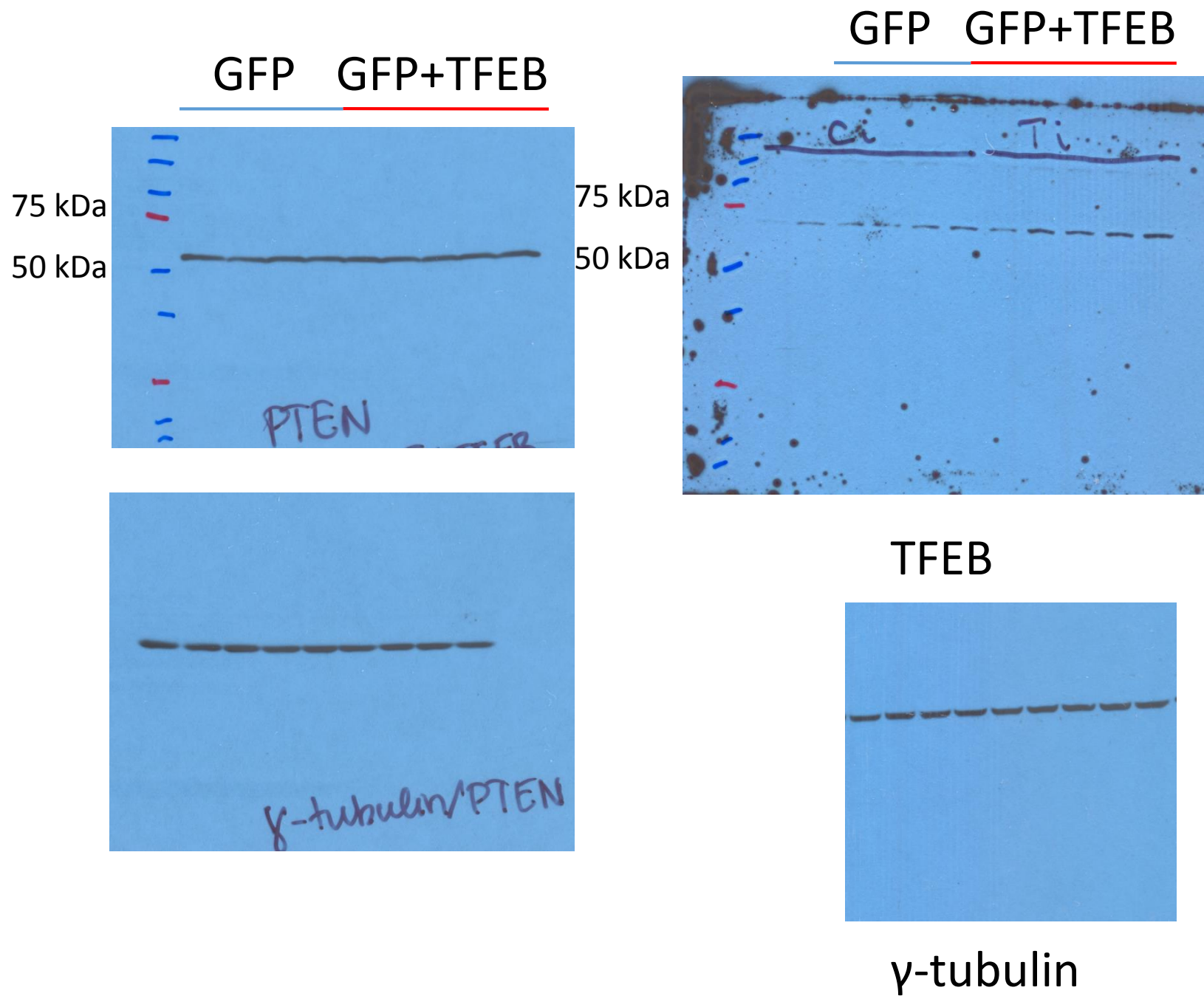

# Supplementary Figure 9 C

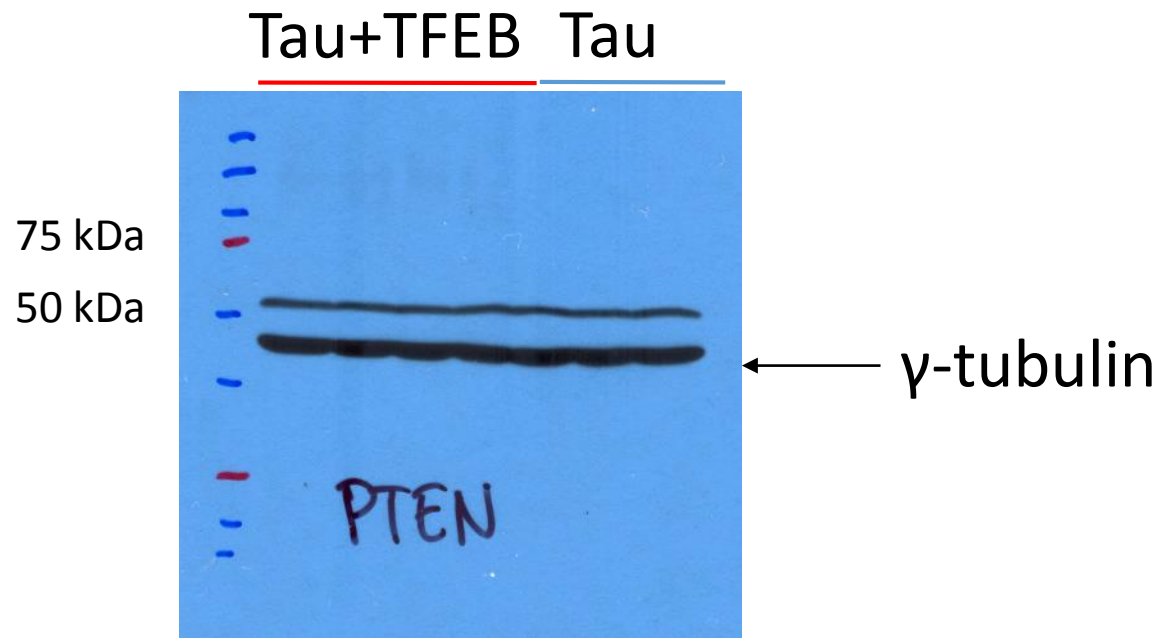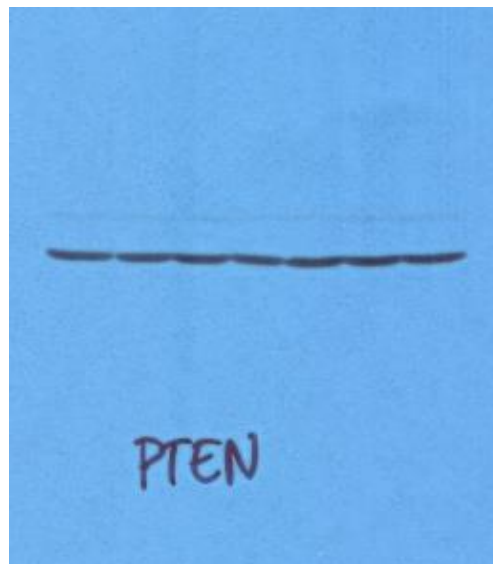

$\gamma$ -tubulin

Supplement: Supplementary file 3 — Source data for Supplementary Figure S9 A C [file emmm0006-1142-SD3.pdf]

Figure 1C

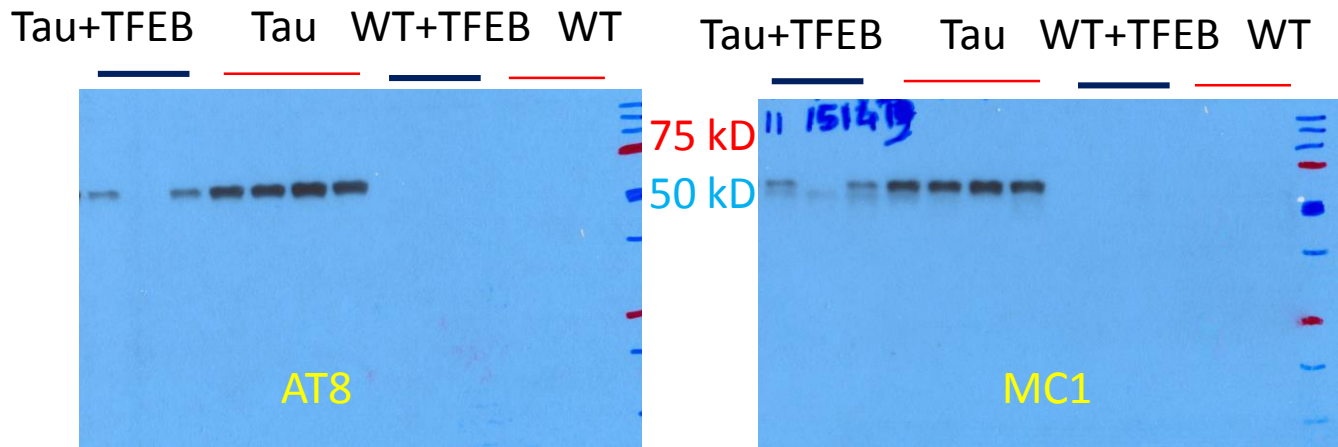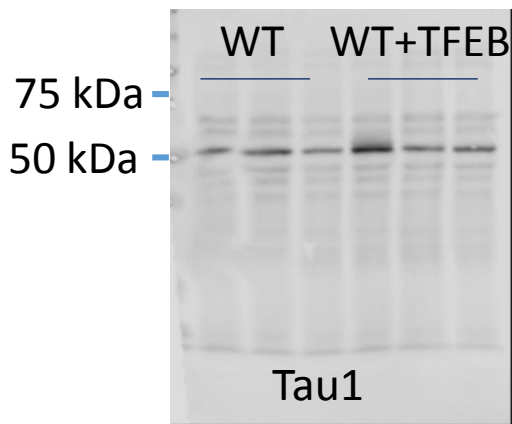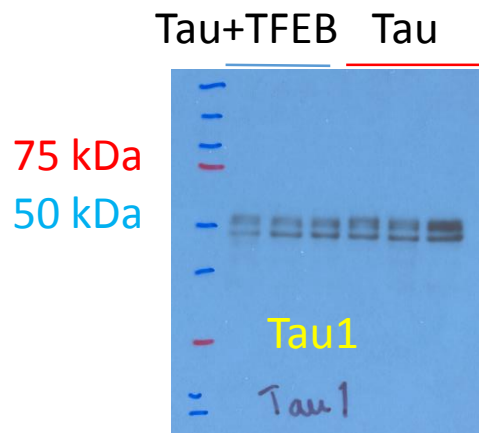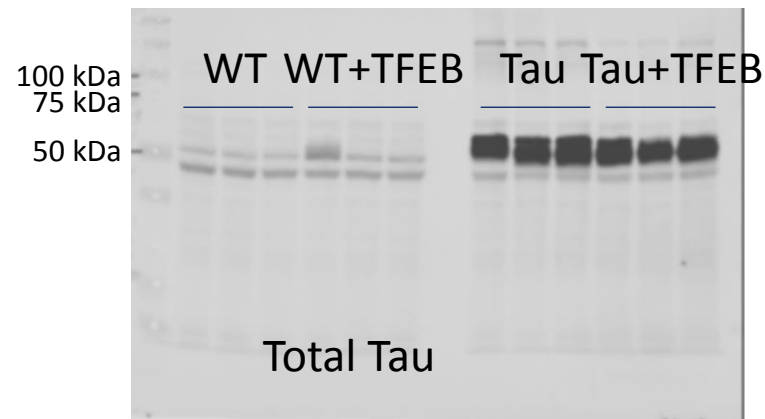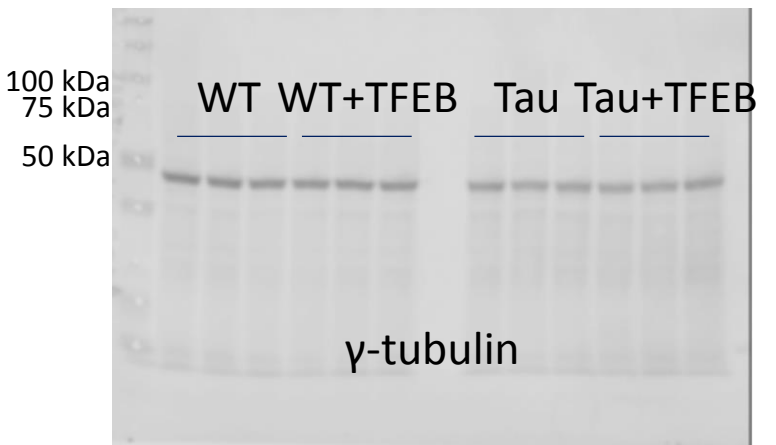

Figure 1D

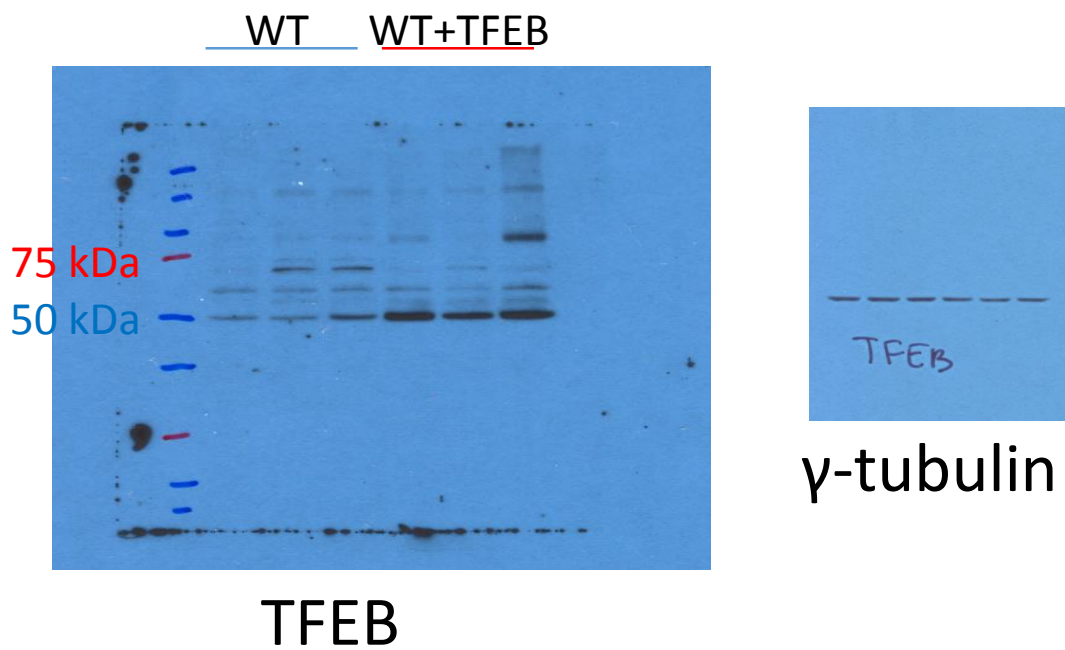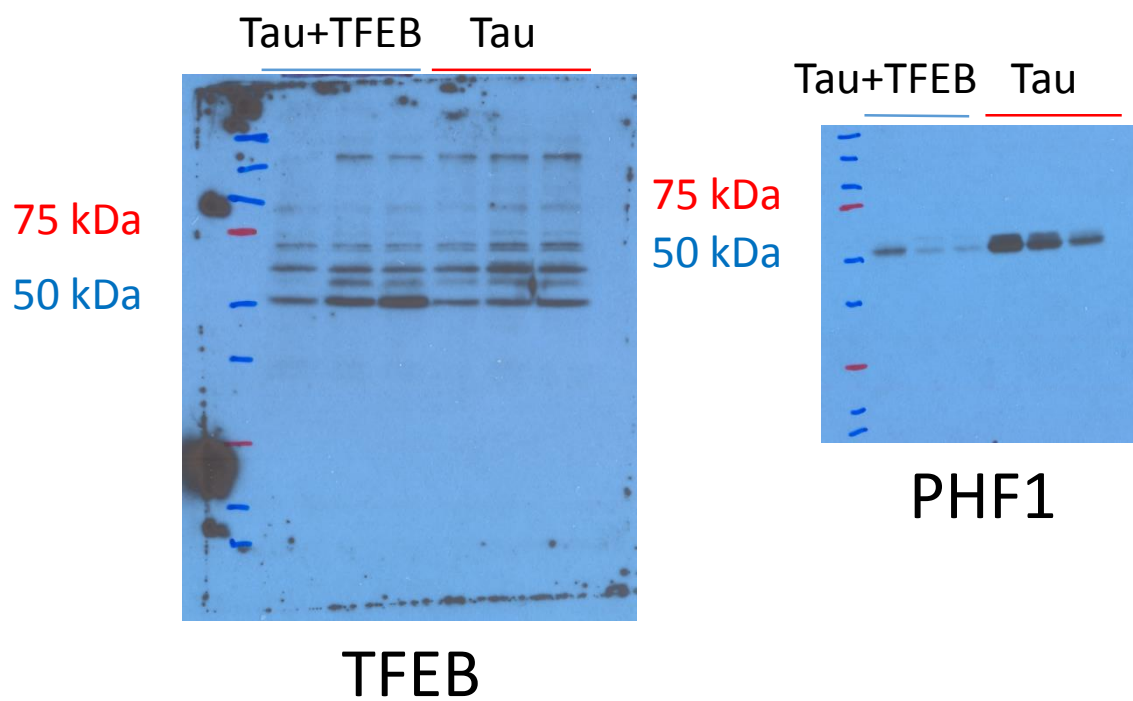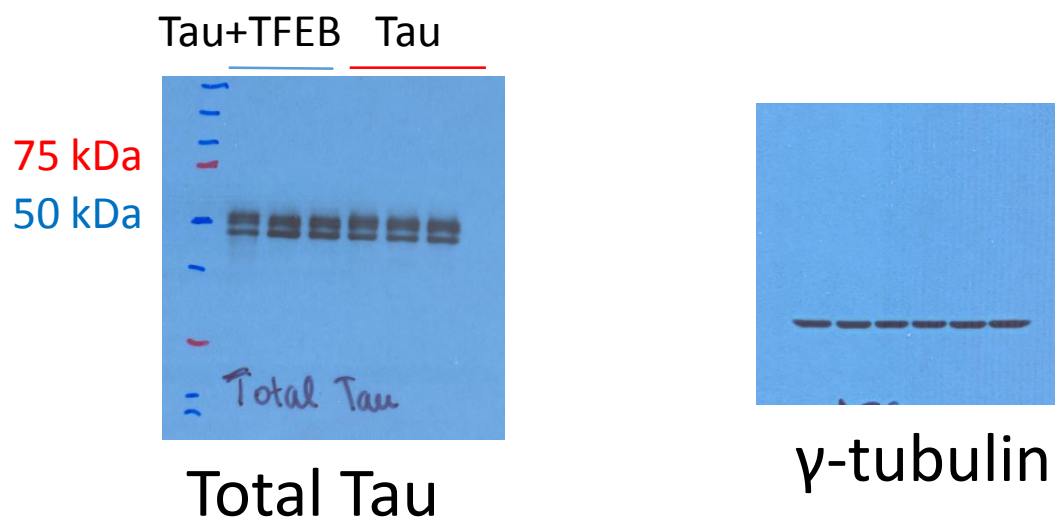

Supplement: Supplementary file 5 — Source data for Figure 1 C D [file emmm0006-1142-SD5.pdf]

Figure 2A

Soluble

Insoluble

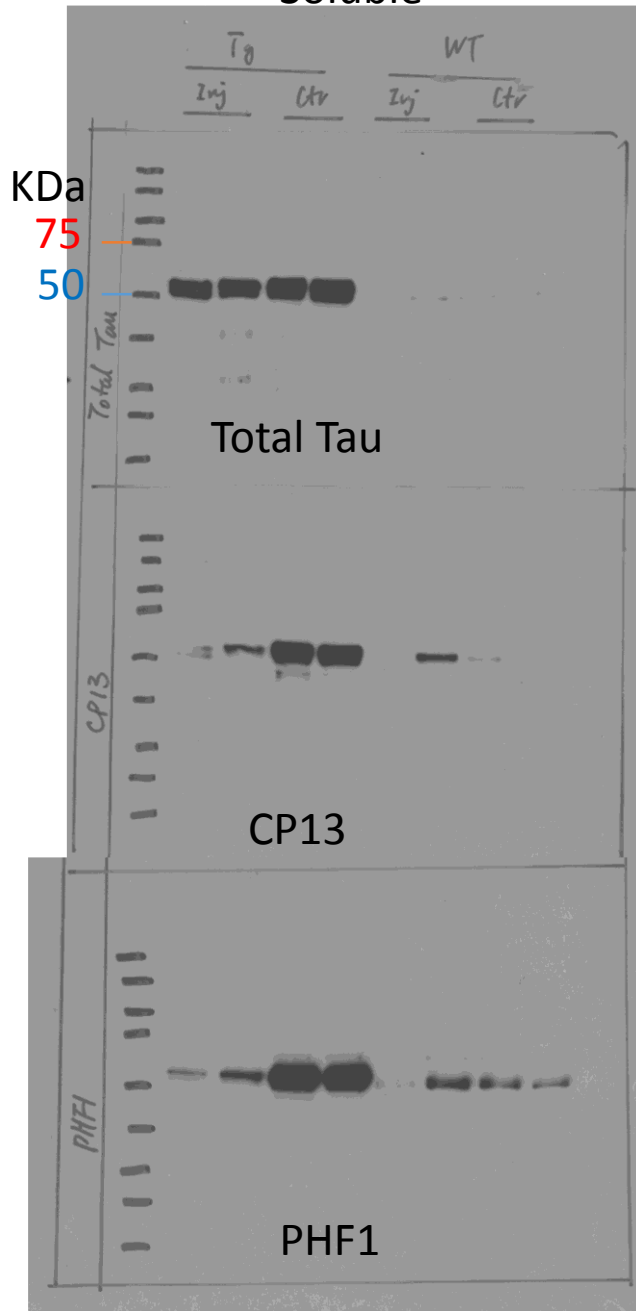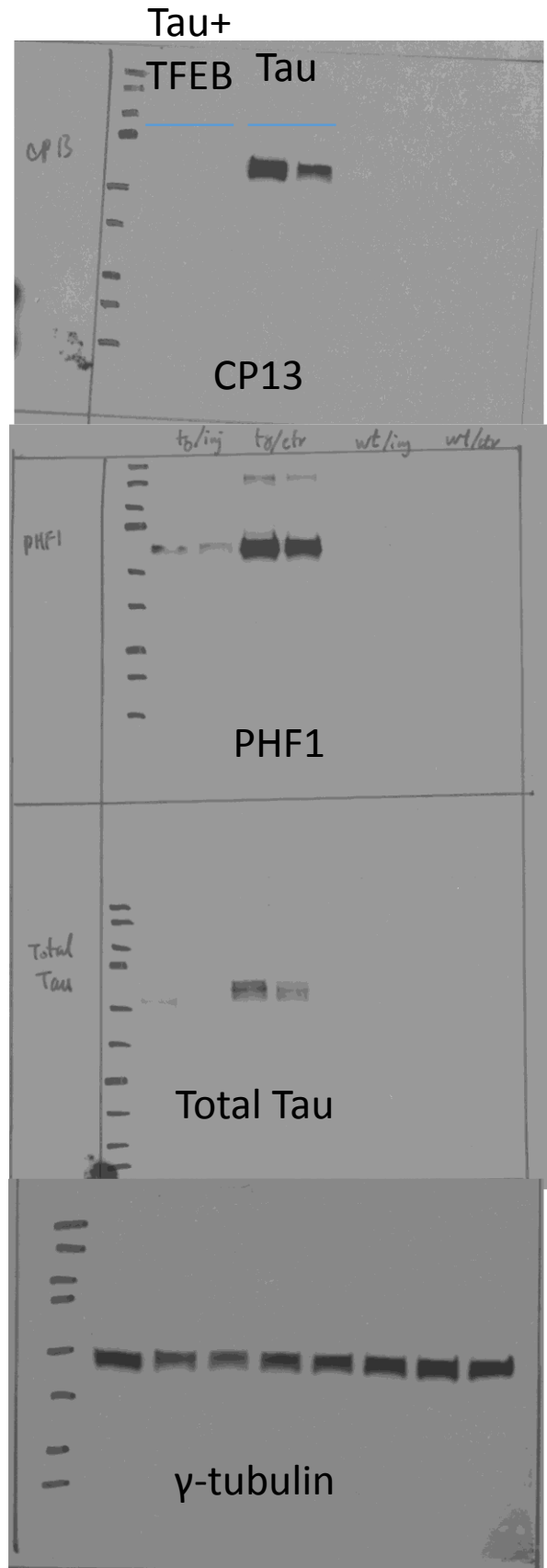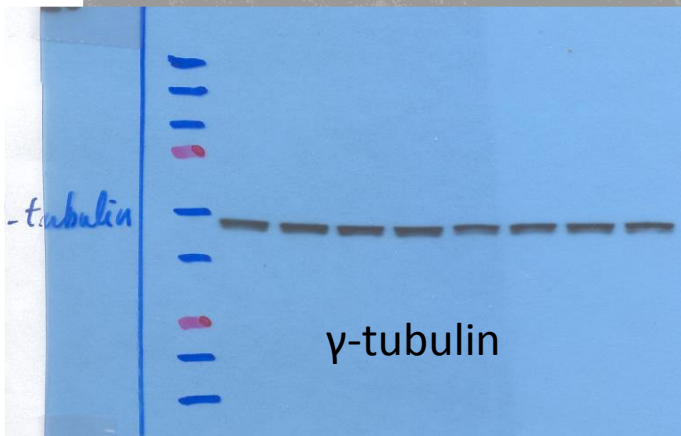

Figure 2B

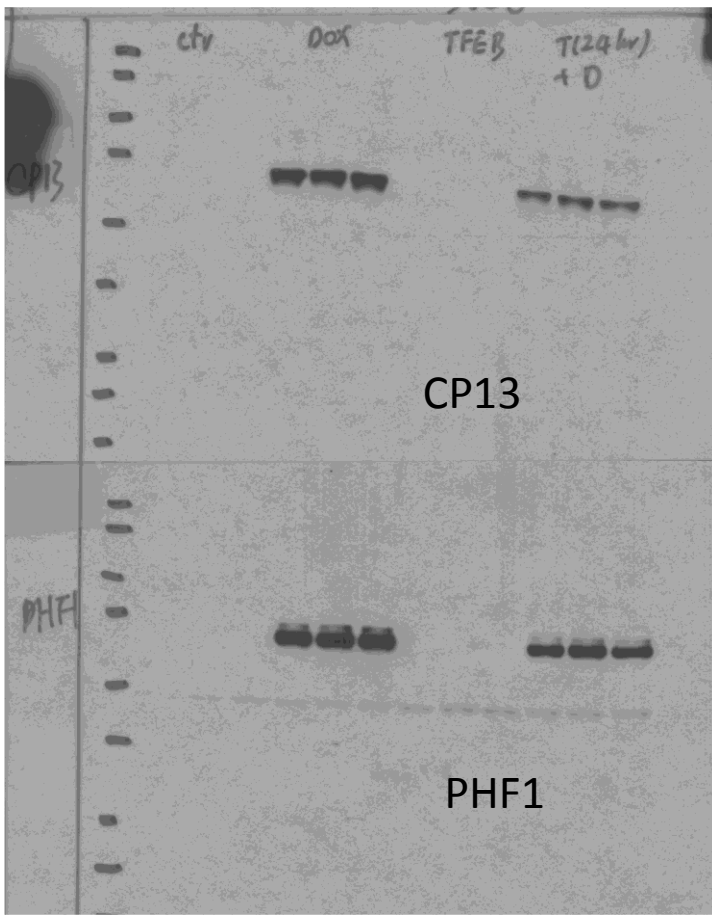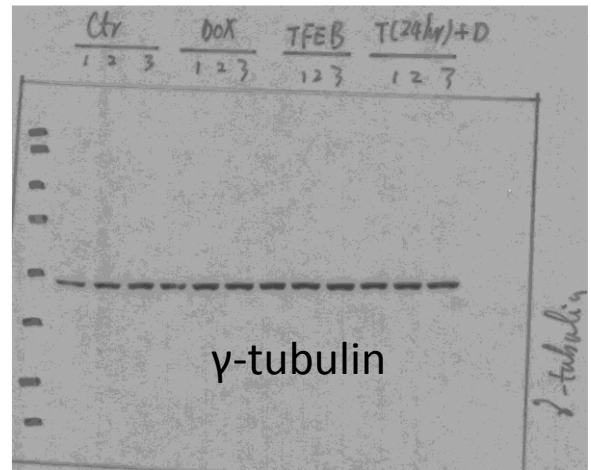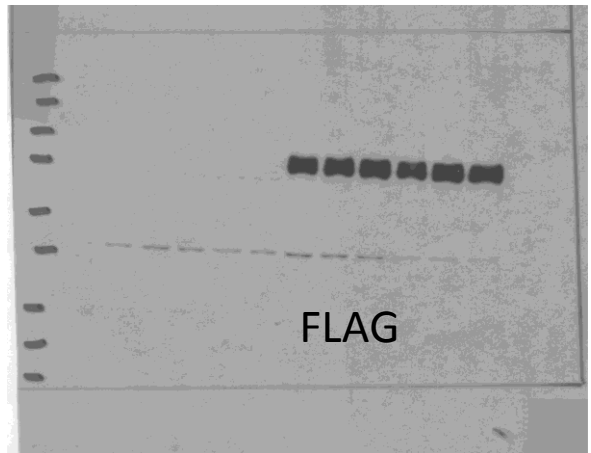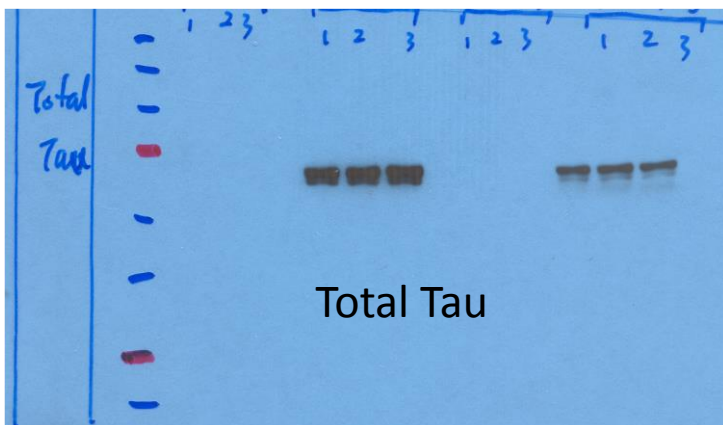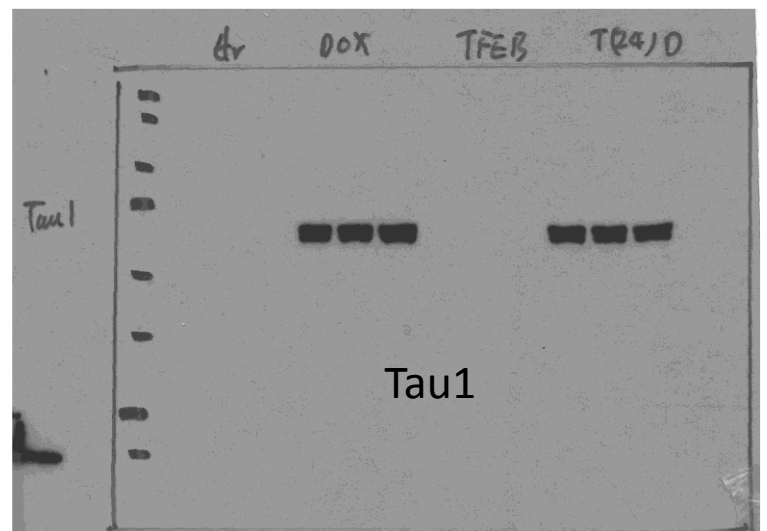

Supplement: Supplementary file 6 — Source data for Figure 2 A B [file emmm0006-1142-SD6.pdf]

Figure 6C

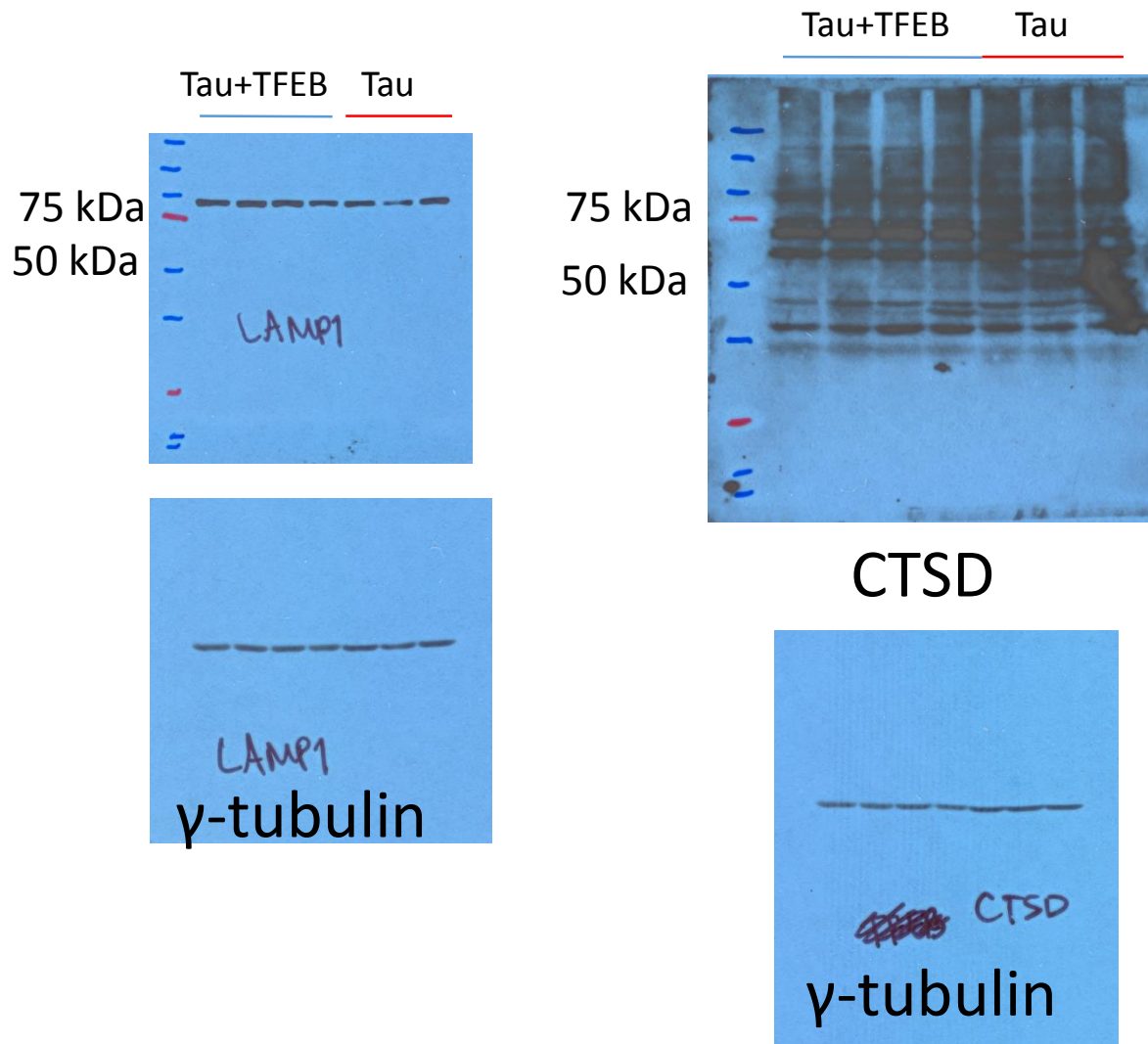

Supplement: Supplementary file 7 — Source data for Figure 6 C [file emmm0006-1142-SD7.pdf]

Figure 7A

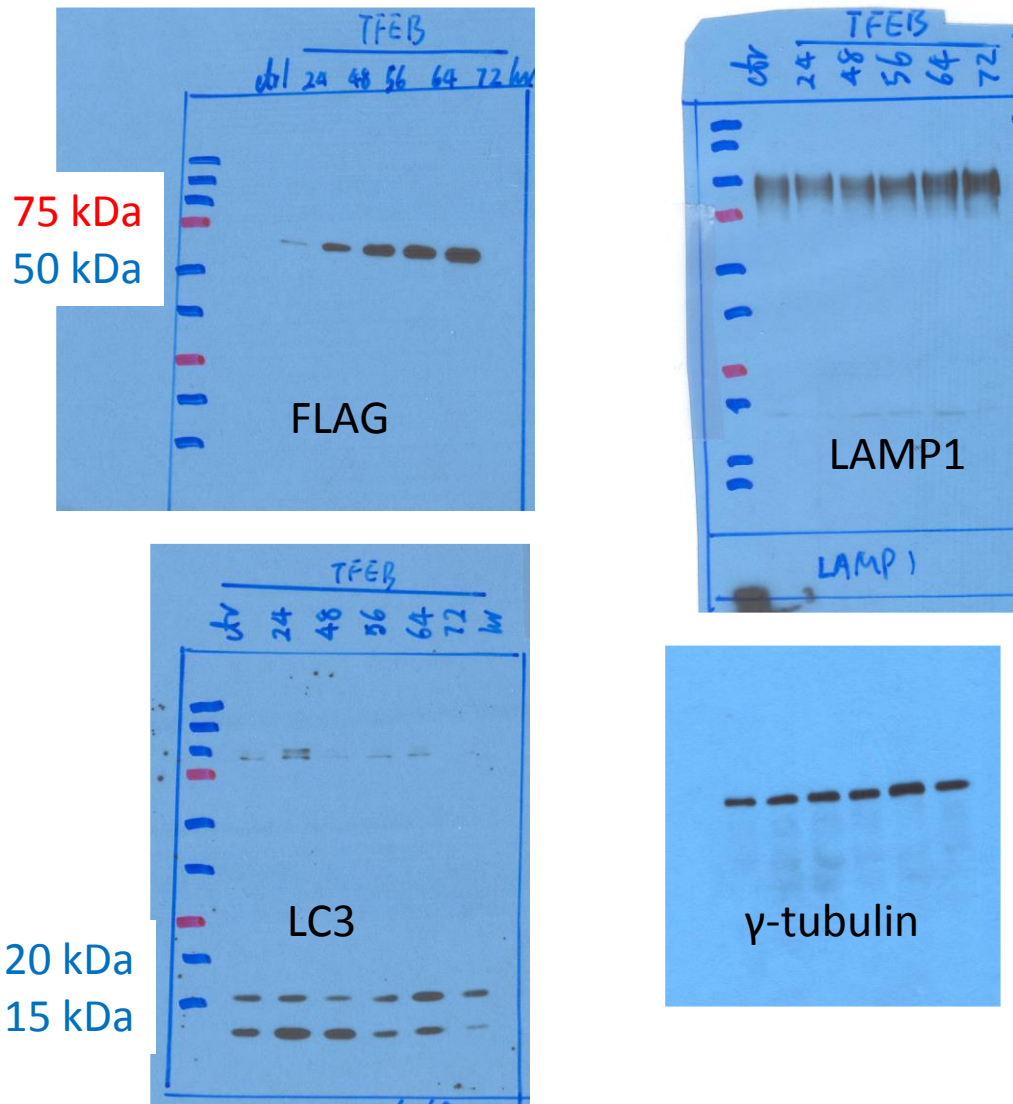

Figure 7D

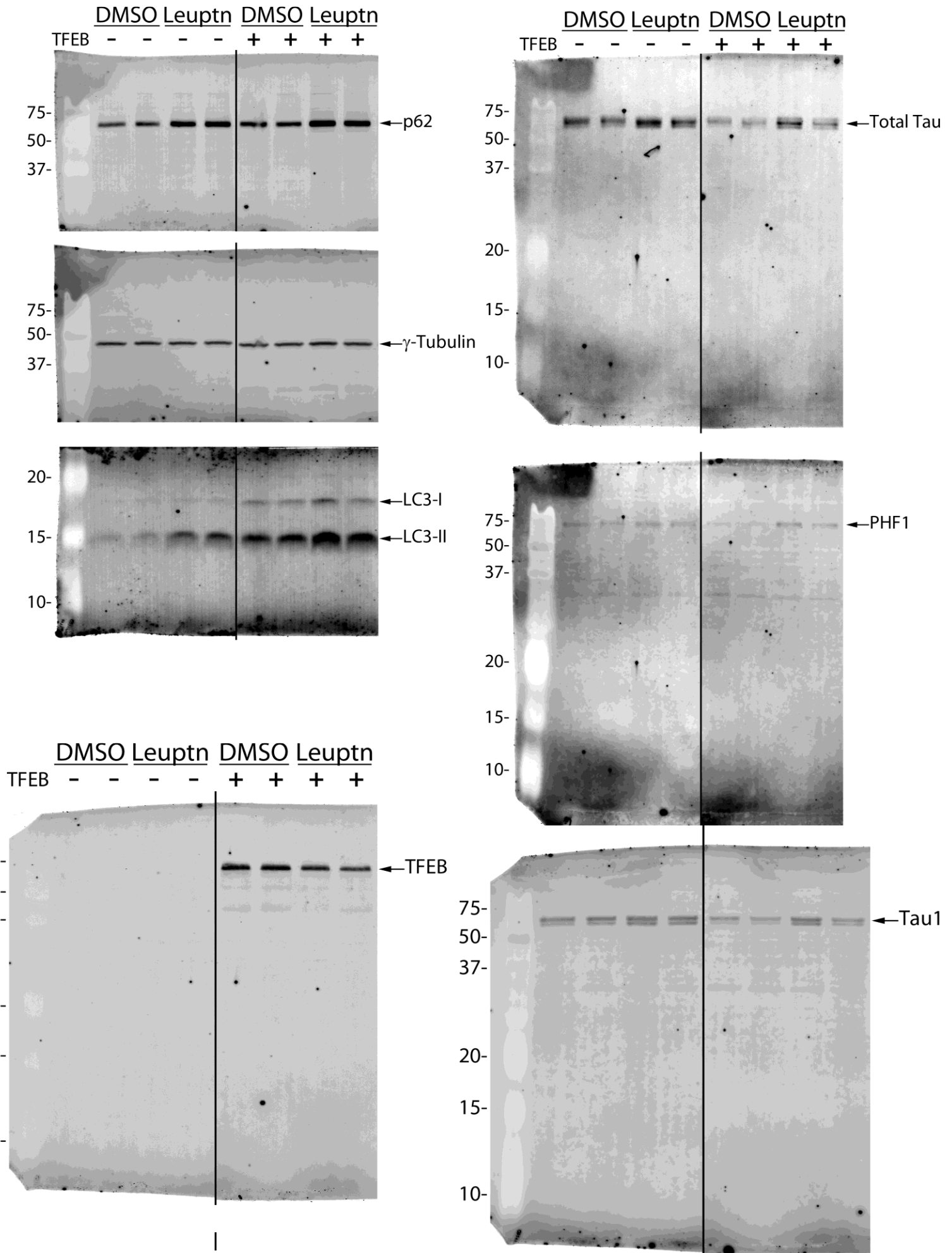

Supplement: Supplementary file 8 — Source data for Figure 7 A D [file emmm0006-1142-SD8.pdf]

Figure 8F

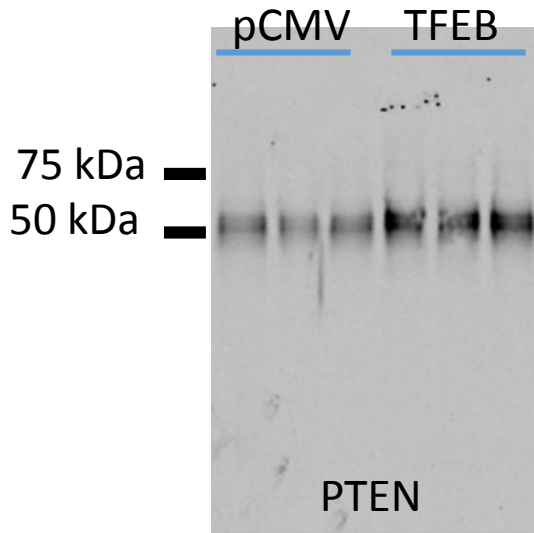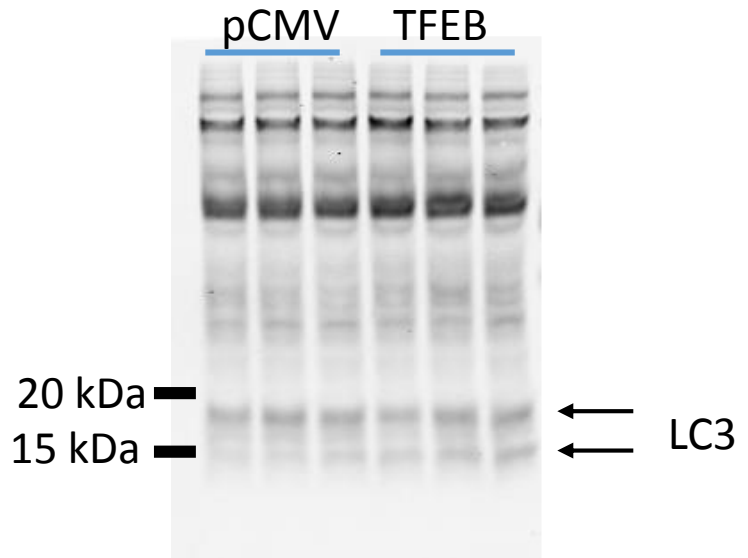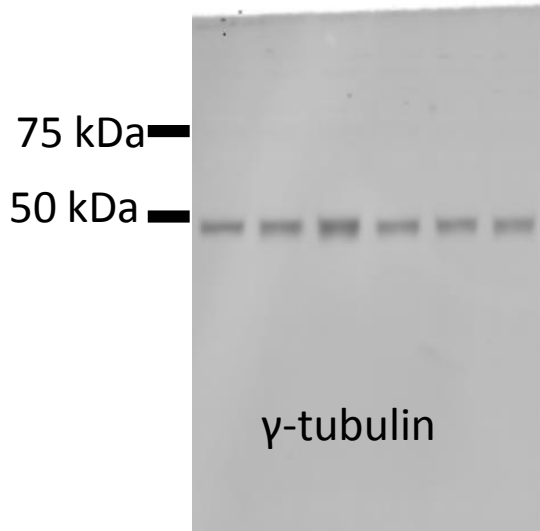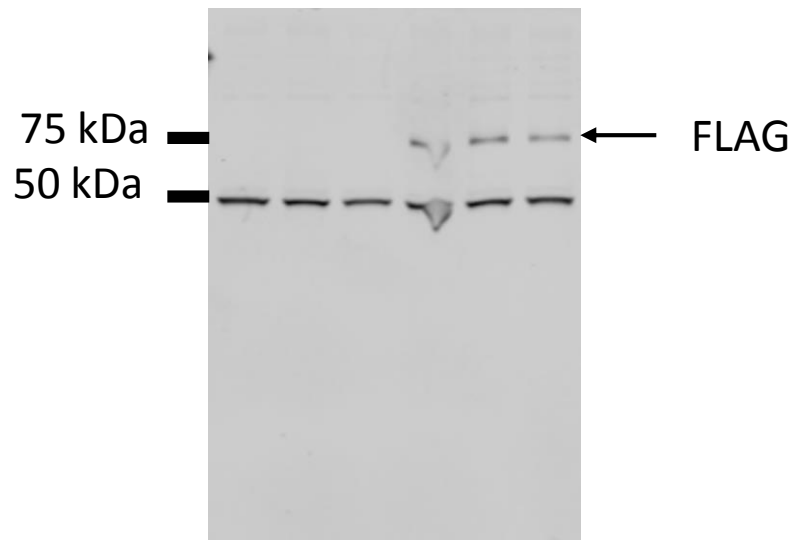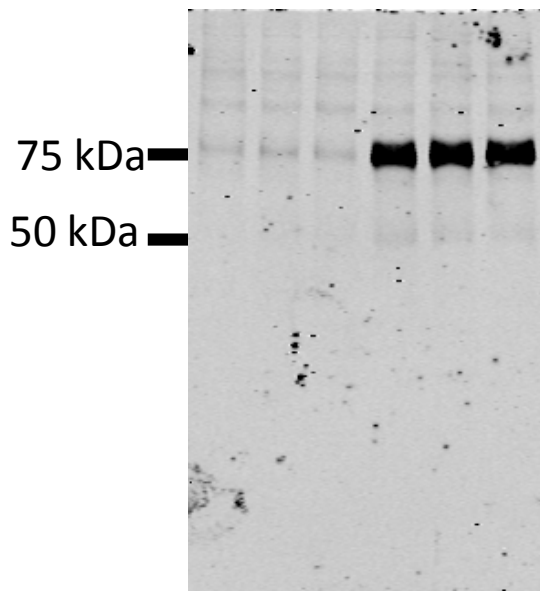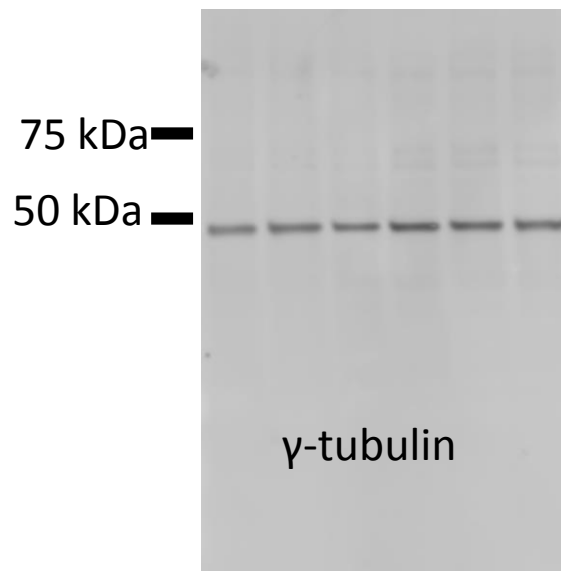

Supplement: Supplementary file 9 — Source data for Figure 8 F [file emmm0006-1142-SD9.pdf]

# Figure 9A

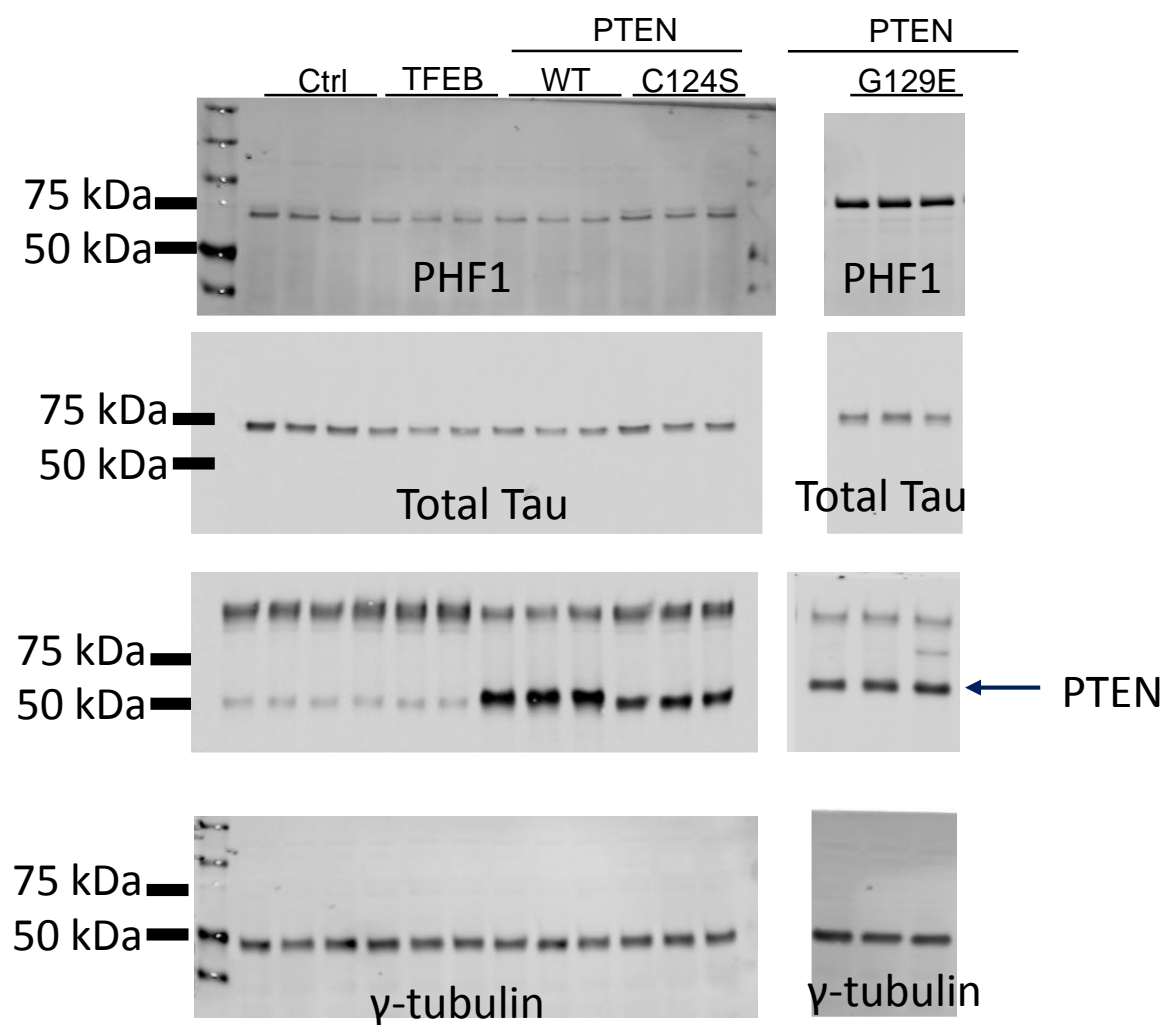

Figure 9D

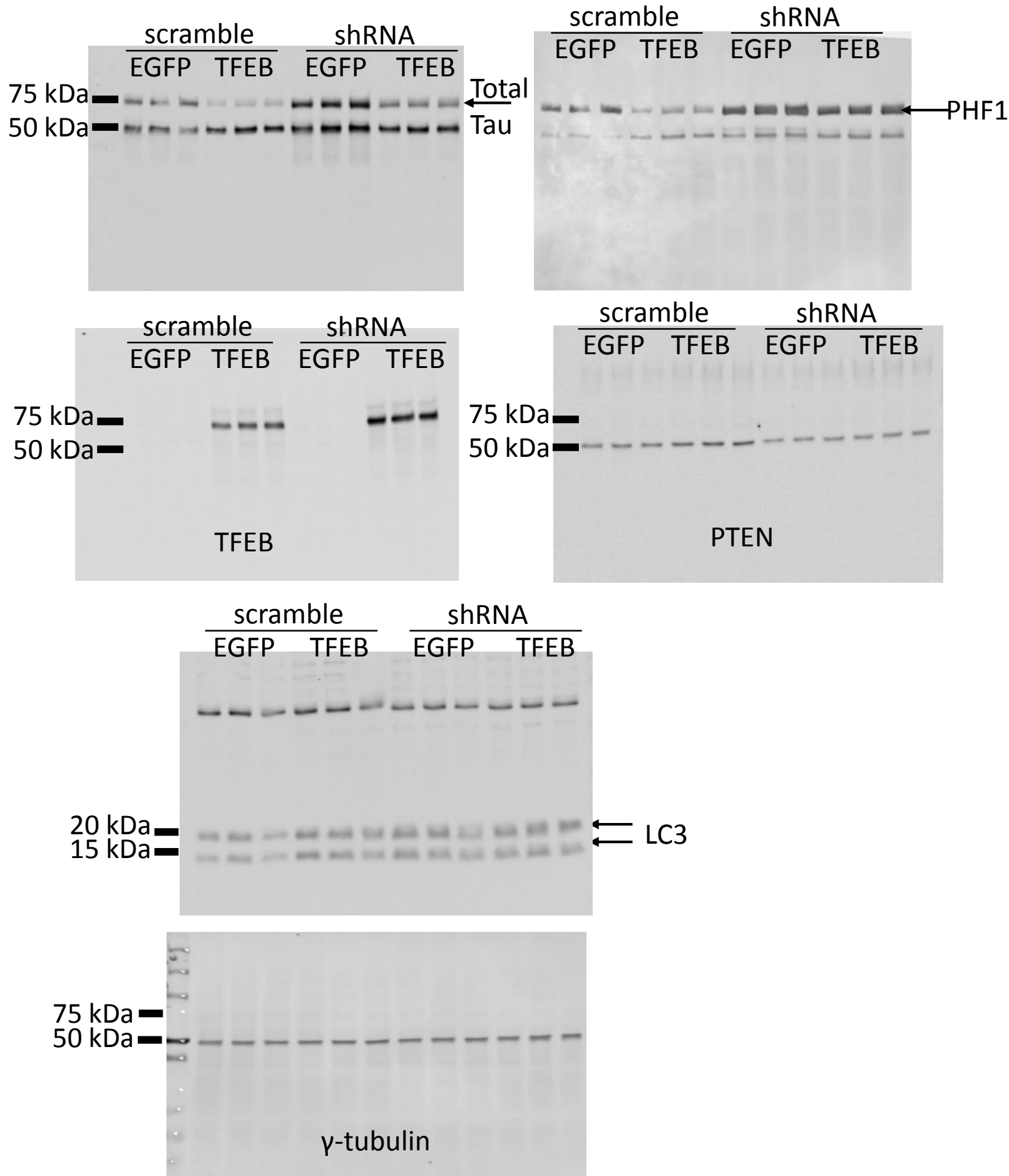

Supplement: Supplementary file 10 — Source data for Figure 9 A D [file emmm0006-1142-SD10.pdf]
